# Cumulative determinants of adolescent health indicators: the effects of social and structural determinants of health and child sexual abuse on overdose and suicide attempt

**DOI:** 10.3389/fpubh.2025.1595115

**Published:** 2025-06-27

**Authors:** Deirdre Colburn, Kimberly J. Mitchell, Ateret Gewitz-Meydan

**Affiliations:** ^1^Crimes Against Children Research Center, University of New Hampshire, Durham, NH, United States; ^2^Faculty of Social Welfare and Health Sciences, School of Social Work, University of Haifa, Haifa, Israel

**Keywords:** image-based sexual abuse, social determinants of health, suicide, child sexual abuse, substance use

## Abstract

**Background:**

Research has increasingly focused on the role of social and structural determinants of health (SDoH) in shaping behavioral and mental health-related outcomes, including suicide and substance use disorder. While child sexual abuse (CSA) is a well-established risk factor for these outcomes, less is known about the association with image-based sexual abuse (IBSA) and its relationship to suicide and overdose risk. Moreover, limited research has explored the interaction between abuse experiences and SDoH deficits, particularly how cumulative disadvantages may exacerbate these risks.

**Methods:**

Using a sample of young adults aged 18–28 (*n* = 2,630) recruited through social media, this study examines the links between SDoH deficits and abuse type prior to age 18 (CSA and IBSA) with suicide attempts and overdose outcomes. Participants reported experiences of hands-on CSA, IBSA, or both, along with self-reported deficits in five key SDoH domains: economic stability, social and community context, housing stability, healthcare access, and neighborhood conditions. A cumulative SDoH deficit scale (0 to 4 + deficits) was created to examine the compounding effects of social and structural disadvantage. Logistic regression models were used to assess the association between SDoH deficits, abuse type, and two primary outcomes: suicide attempts and drug overdose prior to age 18 and lifetime.

**Results:**

The likelihood of each health indicator increased with each additional SDoH deficit reported. The largest percentage-point increase was in lifetime suicide attempt, which was reported by approximately 15.2% of those with 0 SDoH compared to 49.9% of those with 4 or more SDoH deficits. There were also large differences in lifetime suicide attempt by abuse type (20.3% among those with no abuse vs. 60.6% of those with both types). Notably, across each abuse category, the predicted probabilities of suicide attempt, both before age 18 and lifetime, increased with each additional SDoH deficits.

**Discussion:**

Findings suggest that CSA and IBSA do not operate in isolation but interact with social and structural determinants in their association with key health indicators. The results highlight the need for holistic prevention and intervention strategies that address both childhood trauma and modifiable SDoH.

## Introduction

Technology-assisted child sexual abuse, also called technology-facilitated abuse, is a growing concern among law enforcement, practitioners, researchers and policy makers in the United States (1, 2). Image-based sexual abuse (IBSA) during childhood is one important type of technology-facilitated abuse, which includes the non-consensual taking, making, or sharing of sexual images, the forced recruitment of sexual images (“sextortion”), commercial sexual exploitation, or sexual exchanges with an adult (3, 4). Recent research estimates that approximately 16% of US children have experienced at least one type of technology-facilitated abuse before the age of 18, with 11% reporting image-based abuses (5). IBSA can take on many forms, including adult-produced (traditional child sexual abuse material), self-produced, other youth-produced, and non-consensually shared images or videos (4). When combined with “conventional” abuse, or that which traditionally occurs “hands-on” and not exclusively through images or technology, rates of victimization are even higher, raising to more than one-fifth of youth (21.7%) (6). Victims of hands-on child sexual abuse (CSA) and IBSA are at increased risk for adverse mental health outcomes, including suicidal ideation or attempt and substance use disorders. Further, research suggests that IBSA has an independent effect on mental health, even when taking into account CSA and other non-victimization adversities (7).

Child sexual abuse has long been established as a significant predictor of adverse mental health and behavioral outcomes, including increased risks for suicidal ideation, suicide attempts, and substance use disorders (8–11). More recently, attention has expanded to include technology-assisted forms of abuse, particularly IBSA. While hands-on CSA and IBSA share overlapping harms, IBSA uniquely extends the victimization experience into digital spaces, often exposing survivors to ongoing distress and revictimization (12). Despite growing recognition of IBSA as a serious form of child exploitation, research remains limited regarding its impact on long-term mental health outcomes, particularly when compared to conventional hands-on CSA.

To address this gap, the current study explores the intersection of technology-assisted IBSA and hands-on CSA with SDoH, analyzing their cumulative correlation with health indicators – specifically on suicide attempts and overdose risk among adolescents and young adults. By analyzing a large and diverse dataset, we aim to elucidate how structural disadvantages interact with experiences of abuse and how this interaction is related to key health indicators.

### The social determinants of health framework

The social determinants of health (SDoH) framework, which builds upon Bronfenbrenner’s ecological systems theory (1992), has become increasingly prominent in behavioral health research (13–15). This framework emphasizes that health outcomes are shaped not only by biological and genetic factors but also by social and environmental influences. As such, the SDoH framework offers a nuanced understanding of the social contexts that contribute to health behaviors and disparities.

SDoH refers to the structural and systemic conditions that shape health and well-being, including economic stability, access to healthcare, housing security, and neighborhood environments (16). The WHO Commission on the social determinants of health defines SDoH as “the conditions in which people are born, grow, live, work, and age.” These conditions are shaped by families, communities, and the distribution of money, power, and resources at global, national, and local levels, as well as by policy decisions at each of these levels (17). SDoH significantly influences an individual’s ability to meet basic needs such as food security, medical care, and safe living conditions, ultimately shaping health outcomes and contributing to disparities in both physical and mental well-being (18, 19).

Increasingly, research has highlighted the role of SDoH in adolescent well-being (20). Adolescence is a pivotal stage of development marked by biological and neurological changes that influence how young people interact with their families, peers, schools, and health-related behaviors. These shifts significantly shape their future health and life trajectories. Research consistently shows that broader societal structures, such as a country’s economic status, the distribution of wealth, and access to education – all have a profound impact on adolescent well-being. At the same time, nurturing relationships at home, in school, and among peers are essential in supporting adolescents’ growth and facilitating their adjustment into adulthood.

### Social determinants of health, child sexual abuse, suicide, and substance use risk

Cumulative deficits in SDoH have been consistently linked to an elevated risk of mental health disorders, substance use, and suicidality (21–26). These social stressors create chronic adversity, which can erode coping mechanisms, reduce resilience, and increase vulnerability to maladaptive behaviors.

The relationship between SDoH and suicide or substance use is complex and multifaceted. Financial hardship can exacerbate psychological distress, restrict access to mental health care, and contribute to a pervasive sense of hopelessness. Limited healthcare availability delays early intervention for mental health issues, increasing the likelihood of individuals self-medicating with substances. Unstable or unsafe living conditions—including exposure to violence, neglect, or high-risk environments—further intensify stress and emotional dysregulation, making individuals more vulnerable to both substance use and suicidal ideation.

Despite growing recognition of the role of SDoH in shaping health outcomes, research has often examined these factors in isolation rather than in interaction with childhood trauma—particularly experiences of CSA and IBSA. Emerging evidence underscores that the risk for CSA and IBSA is shaped by a convergence of individual, relational, and contextual factors. A meta-analysis focused exclusively on CSA identified prior sexual victimization, familial instability (e.g., presence of a stepfather), and parental dysfunction (e.g., intimate partner violence, mental illness) as the strongest predictors of CSA risk (27). These findings emphasize the ecological and cumulative nature of sexual victimization, particularly in environments marked by compromised caregiving and structural disadvantage. Similarly, Paradiso et al. (28) found that gender-based power imbalances, prior sexual abuse, digital vulnerability, and limited social support significantly increase the risk for IBSA, especially among girls and LGBTQ+ youth. Together, these studies suggest that sexual abuse, whether in childhood or digital contexts, often emerges from conditions of systemic inequity and vulnerability and that these experiences may interact in cyclical, compounding ways. For example, CSA survivors may struggle with trust, emotional regulation, and self-worth—difficulties that, when combined with economic hardship or limited social support, may further intensify mental health challenges. However, the directionality of these relationships remains unresolved, underscoring the need for research that examines the interplay of SDoH and childhood trauma to better inform prevention and intervention strategies.

A more integrative framework is needed to examine how the intersection of CSA, IBSA, and cumulative SDoH deficits shapes long-term mental health trajectories. Adolescents facing both sexual abuse and structural disadvantages may experience compounded risks for severe behavioral health outcomes, including suicide and overdose (8, 9, 11). The accumulation of structural disadvantages likely amplifies the psychological consequences of CSA and IBSA, deepening vulnerabilities and worsening outcomes (29). However, empirical research on these cumulative and interactive effects remains scarce, limiting the ability of healthcare providers, policymakers, and intervention programs to implement comprehensive, evidence-based strategies. This study aims to bridge these gaps by analyzing data from a large, diverse sample of young adults to explore how different forms of child sexual abuse—hands-on CSA, IBSA, and their co-occurrence—interact with cumulative SDoH deficits in predicting suicide attempts and overdose risk during adolescence and young adulthood.

### The present study

CSA and IBSA have both been identified as significant risk factors for adverse mental health and behavioral indicators, including suicidality and substance use disorders (4, 8). While substantial research has examined the long-term effects of hands-on CSA, comparatively less attention has been given to IBSA, despite its overlap with CSA (6) and growing prevalence in digital spaces (3, 12). IBSA introduces unique challenges for survivors, as the permanence and widespread dissemination of explicit images can lead to persistent victimization, ongoing distress, and social isolation (1). Additionally, SDoH—structural and environmental conditions such as economic stability, healthcare access, and housing security—may play a crucial role in shaping adolescent and young adult mental health outcomes (17, 20). Previous studies have demonstrated that individuals facing multiple SDoH deficits are at heightened risk for depression (30), suicidality (22), and substance use (21). However, research has primarily treated SDoH as an independent predictor of adverse health outcomes rather than examining how it interacts with childhood trauma, particularly CSA and IBSA. Understanding the intersection of these factors is essential for identifying the most vulnerable populations and informing targeted interventions.

This study seeks to fill these critical gaps by exploring how cumulative SDoH deficits and different forms of child sexual abuse (hands-on CSA, IBSA, or both) jointly are associated with risk of suicide attempts and drug overdose prior to age 18 and during their lifetime. Using data from a large, diverse sample of young adults (ages 18–28; *n* = 2,630), we construct a cumulative SDoH deficit scale encompassing five domains: economic stability, social and community context, housing stability, healthcare access, and neighborhood environment. We also categorize abuse experiences into four groups: no abuse, hands-on CSA only, IBSA only, and both hands-on CSA and IBSA. Specifically, we hypothesize that: (1) Higher SDoH deficits will be associated with increased risk of suicide attempts and overdose, independent of CSA or IBSA history; (2) Experiencing both hands-on CSA and IBSA will be associated with the highest risk of suicide attempts and overdose compared to either form of abuse alone; (3) The combination of multiple SDoH deficits and CSA/IBSA will have a compounding effect on suicide and overdose risk, with the highest predicted probabilities observed among those experiencing both abuse types and four or more SDoH deficits. In a more exploratory manner, we examine how different SDoH domains contribute uniquely to risk.

## Materials and methods

### Participants and procedure

Data were collected for the *Digital Life Study* through an online questionnaire designed to oversample sexual and gender minority (SGM) individuals. We recruited individuals aged 18–28 across the U.S. through advertisements on Facebook and Instagram. If a prospective participant clicked on the “Learn more” button on the advertisements, they were linked to a secure screener survey hosted by Qualtrics survey platform. Eligibility included being age 18–28, living in the U.S., having English proficiency, and endorsing potential IBSA screening items (explained below). Those who were deemed eligible in the screener survey were redirected to the main behavioral survey, also in Qualtrics.

Our sample was not designed to be nationally representative, as the goals of the larger study were to maximize the number of participants who had experienced IBSA prior to age 18. However, our sample was diverse in terms of urbanicity (26.6% small town or rural area, 38.8% suburban area next to a city, 28.6% urban or city area). To maximize the number of participants with IBSA experience prior to age 18, we used two items in the screener survey: “On a scale of 1 to 5, how likely would you be to…” (1) “Send a photo or video that was sexual?” and (2) “Have someone ask you for a photo or video that was sexual?” Initially, those who answered a likelihood of “2” to above on either of the screener questions were directed to the full survey. Responses were presented to participants on a sliding scale of 1 (Not at all likely) to 5 (Extremely likely) (values labels were not provided for “2,” “3” or “4”). This criterion was modified throughout recruitment to maximize the number of participants with IBSA involvement (i.e., increasing the criteria to answering “3” or above to either of the screener items). Most participants (80%) in the analytic sample were recruited under the “2” + threshold, while only 20% were enrolled after increasing the threshold to “3” or higher.

The analytic sample includes *n* = 2,630 participants who had survey data on key measures that were added to the survey about half-way through recruitment (including SDoH items before age 18, suicide attempt prior to age 18, and overdose prior to age 18). The larger study included *n* = 6,204 participants who completed the survey between June 28, 2023 and April 1, 2024. In the data cleaning process for the larger study, cases were dropped due to reasons such as declined consent (*n* = 227), duplicate or suspected fraud (*n* = 289), incomplete response (*n* = 1729), or had no matching screener data (*n* = 161). To screen out any suspect or extreme responses, a research assistant on our team checked open-ended qualitative incident narratives provided by the respondent (“In a few sentences, please describe what happened.”). Any that were deemed as “suspect” were flagged and reviewed. Cases were considered complete if they reached the 90% Progress threshold determined by Qualtrics survey platform. There were 20,795 completed screener surveys, yielding 6,204 full valid surveys, indicating a completion rate of 30%. The survey took about 25 min to complete. Once complete, participants were entitled to a $15 Amazon gift card, mailed to valid U.S. non-P. O. box mailing addresses, as a thank you for their time. All participants provided consent for their participation. The study was reviewed and approved by the University of New Hampshire Institutional Review Board

### Measures

#### Demographics

We asked several questions to collect information on participant age, sex at birth, sexual identity (any sexual minority identity vs. no; 2.6% missing data), gender identity (any gender minority identity vs. no), race (any racial minority identity vs. no), and ethnicity (Hispanic/Latino ethnicity vs. non-Hispanic). Supplementary Table 1 contains the demographic characteristics of our analytic sample.

#### Abuse type

Participants were asked a series of questions detailing their experience with several types of child sexual exploitation before the age of 18. Endorsement of *any* of the following five IBSA items constituted this form of victimization:

**Non-Consensual Taking or Making or Sharing**: Participants were asked, “Before the age of 18, did someone ever take, make, or share with other people a sexual picture or video of you without your permission?” This was designed to measure both the taking of sexual images and making, through photoshop or photo editing, sexual images of the youth without consent.
**Forced or Pressured Production**: “Before the age of 18, did someone ever threaten, try to force you, or strongly pressure you to provide sexual pictures or videos online or through a cell phone?”
**Threatened Sharing:** This screener asked “Before the age of 18, did someone ever threaten to share a sexual picture or video of you to get you to do something – like take or send other sexual pictures of yourself, have a sexual relationship with them, pay them money, or something else?”
**Self-Produced Image Sharing with Someone Older**: This screener was designed to measure a sexual relationship with an adult 5 or more years older than the youth. The question asked, “Before the age of 18, did you ever share sexual pictures or videos (online or through a cell phone), even if you wanted to, with a person who was 5 or more years older than you?”
**Commercial Sexual Exploitation**: This item was designed to measure sexual exchanges of a commercial nature. “Before the age of 18, did you ever make, send or post sexual pictures or videos of yourself over the Internet or a cell phone (including texting) in exchange for money, drugs, or other valuable items?”

Hands-on child sexual abuse (CSA) was measured using two items in the survey: “At any time in your life before age 18, did a grown-up you knew touch your private parts when they should not have or make you touch their private parts? Or did a grown-up you knew force you to have sex?” This question was repeated for a grown-up you did not know. Response options to both questions included no, yes, or prefer not to answer. Respondents who said yes to either question were coded as having experienced hands-on sexual abuse before age 18.

An abuse type variable was created using the items above. Participants were coded into one of four categories: (1) IBSA only if they endorsed IBSA but *not* hands-on CSA; (2) hands-on abuse only if they endorsed hands-on CSA but *not* IBSA; (3) none if they did not endorse either; or (4) both if they endorsed both abuse types.

#### Social determinants of health

Social determinants of health screening tools are designed primarily for in-person assessment of adults in clinical settings. To measure SDoH deficits, before the age of 18, items and scales were drawn from previous work and modified and piloted with youth in a similar study (31). Items cover five domains of SDoH, detailed below:

##### Economic stability

This was captured with three items including self-reported difficulty (for you or your family) paying bills, having your cell phone shut off, or skipping meals before the age of 18 (each on 5-point scale ranging from 0 (never) −4 (always)).

##### Social and community context

***Lifetime non-victimization adversity*** due to non-violent traumatic events and chronic stressors before age 18 was measured using 11 yes/no items (for example, serious illnesses, family homelessness) (32). Items were summed to reflect a total adversity count.

***Discrimination*** was measured using nine items from the Everyday Discrimination Scale (33). Response options ranged from 0 (never) to 5 (almost every day). Examples include being treated with less courtesy and respect than others to being threatened or harassed before age 18 (*α* = 0.63).

##### Health care

###### Dental visit

Participants were asked to report how often they went to the dentist before age 18. Response options ranged from “Once a year” to “I never went to the dentist before I turned 18.” Responses were dichotomized into “no deficit (0)” vs. “deficit (1)”; those who reported going to the dentist less than once every 4–5 years before age 18 were coded as 1.

##### Housing stability

###### Unstable housing

Five items covered alternative sleeping situations because participants did not have a permanent place to stay before age 18 (34). Participants were asked if they experienced any of the following sleeping situations (select all that apply): sleeping outside, in a car, or somewhere else not meant for sleeping (like a park, laundromat, abandoned house); sleeping in a motel, hostel, or hotel when you were not on vacation; sleeping in an emergency or homeless shelter; couch surfing or staying at somewhere else’s place for a while; sleeping somewhere else because they did not have a permanent place to stay (like an emergency room, jail, or detention facility) (*α* = 0.73). Responses options were yes/no and the total number endorsed was summed to create a total unstable housing deficit score.

##### Neighborhood and built environment

*Physical home environmental conditions*. Eight items cover problems where participants lived (35). Participants were told to think about their permanent place of residence for the majority of their life before age 18: bugs everywhere, mold, lead paint or pipes, not enough heat, the oven or stove does not work, there are no smoke detectors or they do not work, water leaks, no or frequent loss of electricity (*α* = 0.74). Response options for each were yes/no and summed to create a total.

*Physical neighborhood environmental conditions*. Twelve items measure perceptions of the severity of different neighborhood conditions (36). Participants were asked to rate each one as to whether it is: no problem, a minor problem or a serious problem in the neighborhood they lived before the age of 18: gangs, graffiti, drugs, homelessness, buildings with broken windows or other damage, abandoned or boarded up buildings, violent crime, police or ambulance sirens at night, gun shots at night, fighting, trash or litter, break-ins or burglaries (*α* = 0.93). Those who responded with a minor or serious problem were coded as 1 (yes) for each of the items, while all others were coded as 0 (no).

A total SDoH deficit score was created using each of the domains above. A “high” score in each of the domains above (defined as greater than or equal to 1 standard deviation above the mean, except in the case of dental visits as explained above) counted as one point toward the total SDoH deficit sum score. SDoH deficit scores ranged from 0 to 9. For analyses, we collapsed this into 0, 1, 2, 3, or 4 + deficits. Supplementary Table 2 shows original item question wording, mean (SD), range, and new dichotomized item threshold.

#### Suicide & overdose health indicators

##### Suicide attempt

Both lifetime and before age 18 suicide attempts were measured. Participants were asked “Have you ever tried to die by suicide?” (yes/no). Those who said yes were then asked, “Did you try to die by suicide before you turned 18 years old?” (yes/no).

##### Drug overdose

Lifetime and before age 18 drug overdose were measured. Participants were asked “Have you ever overdosed on medication, pills, or other drugs so that you got really sick and had to go to the hospital?” (yes/no). Those who said yes were then asked about before the age of 18 (yes/no).

### Data analysis

Data were analyzed using Stata/SE version 17.0. Data were weighted to general population targets from the Current Population Survey for 18–28 year olds on age, gender, education, race and Hispanic ethnicity, as well as gender and sexual identity from Pew Research Center (37, 38).^1^ Weights were applied in bivariate and multivariate analyses. Missing data were less than 5% and conservatively coded as “no” for dichotomous variables.

First, to examine differences in SDoH deficits by abuse type, we use bivariate analyses to report the endorsement of each SDoH deficit, as well as total deficit count, among those reporting each type of abuse. Overall Chi-squared test statistics were reported. We also examined pairwise comparisons (i.e., differences in endorsement of SDoH domains between each level of abuse – CSA vs. none, IBSA vs. none, both vs. none).

Next, we analyzed the endorsement of our outcome variables, any or before 18 suicide attempt and any or before 18 overdose, for SDoH deficit count and abuse type with overall Chi-squared tests. Pairwise comparisons were again used to compare endorsement of each outcome by each level of SDoH deficit (i.e., 1 vs. 0, 2 vs. 0, 3 vs. 0) and abuse type (i.e., CSA vs. none, IBSA vs. none, both vs. none).

Using logistic regressions, we calculated the predictive margins of each outcome based on the intersection of the number of SDoH deficits and abuse type. We repeat these analyses at the intersection of SDoH domain and abuse type. All models controlled for current age, sexual minority identity before age 18, gender minority identity before age 18, racial minority identity, ethnic minority identity, and sex at birth. Statistical significance threshold was set at *p* < 0.05.

## Results

### Sample characteristics

Nearly 40% of participants reported experiencing only IBSA before age 18 (38.3%), 12.4% experienced both CSA and IBSA, and 3.7% experienced CSA only. About half (45.7%) did not report any abuse. Given our method of oversampling for IBSA victimization, it is not surprising that rates of IBSA are higher than nationally representative samples (5). Rates of lifetime and before age 18 suicide attempt were higher than national estimates (29.6 and 25.0% respectively), though national estimates, such as that from the Youth Risk Behavioral Survey, often use different timeframes for attempt history (i.e., past 12 months vs. lifetime) (39, 40). Approximately 8.8% of our sample reported lifetime drug overdose, and 5.1% reported overdosing on drugs before age 18 (Table 1). Overdose rates were also higher than national estimates, though estimates typically rely on emergency department data rather than self-report and may not be comparable (41).

**Table 1:** Abuse and behavioral health indicators among an online sample of young adults (*n* = 2,630).

Construct	*n* (unweighted %)
Abuse type	
None	1,203 (45.7)
CSA only	96 (3.7)
IBSA only	1,006 (38.3)
Both CSA and IBSA	325 (12.4)
Suicide attempt	
Lifetime	778 (29.6)
Before age 18	658 (25.0)
Overdose	
Lifetime	230 (8.8)
Before age 18	135 (5.1)

CSA, child sexual abuse. IBSA, image-based sexual abuse.

### Bivariate analyses

#### SDoH by abuse type

Table 2 shows SDoH deficit endorsement by abuse type. Overall, our sample reported an average of 1.17 (SD = 1.71) SDoH deficits. Mean SDoH deficit count varied by abuse type: mean deficit count was 2.89 (SD = 2.52) among respondents who endorsed both abuse types, 1.30 (SD = 1.70) among those with IBSA only, 1.06 (1.49) among those with CSA only, and less than one (0.76 (SD = 1.27)) among those who did not endorse any abuse types (*p* < 0.001).

**Table 2:** Individual and cumulative social and structural determinants of health deficits for youth based upon CSA type experienced before age 18.

SDoH deficits	All (*N* = 2,630)	Abuse type				*p*-value
		None (*n* = 1,203)	Hands-on CSA Only (*n* = 96)	IBSA Only (*n* = 1,006)	Both CSA and IBSA (*n* = 325)	
	*n* (weighted %)					
**Economic stability prior to age 18**						
Not enough money to pay the bills	468 (15.4)	126 (10.7)	25 (15.3)	193 (17.0)^a^	124 (34.7)^a^^,^^b^^,^^c^	<0.001
Cell phone turned off	375 (13.8)	93 (10.1)	17 (15.7)	163 (14.7)	102 (29.1)^a^^,^^c^	<0.001
Skipped meals	548 (16.9)	125 (8.3)	24 (12.5)	239 (21.4)^a^	160 (46.2)^a^^,^^b^^,^^c^	<0.001
**Social context prior to age 18**						
Non-victimization adversity	452 (13.0)	100 (7.3)	20 (8.2)	193 (14.2)^a^	139 (40.6)^a^^,^^b^^,^^c^	<0.001
Discrimination	500 (14.9)	119 (8.4)	26 (16.9)	222 (19.3)^a^	133 (31.5)^a^^,^^c^	<0.001
**Housing stability prior to age 18**						
Unstable housing	163 (4.5)	32 (2.9)	3 (1.3)	63 (4.4)^a^	65 (14.6)^a^^,^^b^^,^^c^	<0.001
**Healthcare prior to age 18**						
Saw dentist <1/4–5 years	343 (12.0)	110 (9.0)	19 (11.7)	131 (11.8)^a^	83 (28.9)^a^^,^^b^^,^^c^	<0.001
**Neighborhood and built environment prior to age 18**						
Residence problems	404 (11.8)	115 (8.0)	16 (7.1)	159 (12.9)^a^	114 (28.9)^a^^,^^b^^,^^c^	<0.001
Neighborhood problems	450 (15.0)	123 (11.7)	17 (17.4)	179 (14.3)	131 (34.8)^a^^,^^c^	<0.001
**Number SDoH deficits**						**<0.001**
0	1,237 (50.8)	728 (60.1)	34 (56.5)	416 (44.8)	59 (23.5)	
1	534 (21.9)	249 (22.2)	16 (8.9)	227 (23.9)	42 (16.4)	
2	293 (10.7)	109 (8.8)	18 (19.9)	127 (12.3)	39 (11.7)	
3	195 (6.1)	46 (3.8)	13 (8.6)	88 (7.5)	48 (11.3)	
4 or more	371 (10.5)	71 (5.1)	15 (6.0)	148 (11.4)	137 (37.2)	
Cumulative SDoH M (SD)	1.17 (1.71)	0.76 (1.27)	1.06 (1.49)	1.30 (1.70)^a^	2.89 (2.52)^a^^,^^b^^,^^c^	<0.001

SDoH, social determinants of health; CSA, child sexual abuse (conventional, offline), IBSA, image-based sexual abuse (technology).

a Significantly different from none.

b Significantly different from Hands-on.

c Significantly different from IBSA only.

All individual SDoH deficit domain endorsements also significantly differed by abuse type (*p* < 0.001). Overall, 15.4% of the sample reported not having enough money to pay the bills before age 18, 13.8% reported having their cell phone turned off, 16.9% reported having to skip meals, 13.0% reported high non-victimization adversity before age 18, 14.9% reported experiencing high levels of discrimination, 4.5% reported unstable housing before age 18, 12.0% reported having seen a dentist less than 1 time every 4–5 years before age 18, 11.8% reported poor home conditions (e.g., bugs, mold), and 15.0% reported high neighborhood disorder. Approximately 50.8% of the sample did not report any SDoH deficits before age 18; 10.5% reported 4 or more deficits.

Pairwise comparisons found that those with hands-on CSA did not significantly differ statistically from those with IBSA only across individual types of SDoH. Those with both abuse types significantly differed from all other abuse categories across most SDoH domains except for having their cell phone turned off, discrimination, and neighborhood problems, in which those with both types of abuse did not differ from those with hands-on CSA only.

#### Health outcomes by SDoH and abuse type

Table 3 shows the rates of suicide attempt before age 18, drug overdose before age 18, lifetime suicide attempt, and lifetime drug overdose by SDoH deficit count and abuse type. The proportion of those reporting each outcome increased substantially from “0” to “4 or more” SDoH deficits. Twelve percent of those with zero SDoH deficits reported any suicide attempt before age 18, compared to 17.9% with 1 deficit, 25.3% with 2 deficits, 22.9% with 3 deficits, and 41.8% with 4 or more SDoH deficits (*p* < 0.001). The proportion of those with 4 + SDoH deficits reporting suicide attempt before age 18 is significantly different from all other levels of SDoH. Less than 2% of those with zero deficits reported any drug overdose before age 18, followed by 3.3% with 1 SDoH deficit, 2.7% with 2 deficits, 4.4% with 3 deficits, and 11.5% of those with 4 or more deficits (*p* < 0.001). The proportion of those with 4 + SDoH deficits reporting overdose before age 18 was significantly different from all other levels of SDoH. Trends were not restricted to experiences prior to age 18; similar patterns persisted when considering lifetime suicide attempt (15.2% among those with 0 deficits vs. 49.9% among those with 4+) and lifetime overdose (4.6% among those with 0 deficits vs. 20.9% among those with 4+).

**Table 3:** Suicide and overdose across different numbers of SDoH deficits and type of abuse.

Construct	*n*	Suicide attempt before age 18 (*n* = 658)	Overdose before age 18 (*n* = 135)	Lifetime suicide attempt (*n* = 778)	Lifetime overdose (*n* = 230)
		*n* (weighted %)			
No. SDoH deficits					
0	1,237	178 (11.8)	24 (1.7)	222 (15.2)	54 (4.6)
1	534	133 (17.9)^a^	28 (3.3)	168 (23.1)^a^	43 (5.9)
2	293	85 (25.3)^a^	15 (2.7)	95 (28.6)^a^	31 (9.3)
3	195	75 (22.9)^a^	19 (4.4)	83 (25.6)^a^	26 (9.8)
4 or more	371	187 (41.8)^a^^,^^b^^,^^c^^,^^d^	49 (11.5)^a^^,^^b^^,^^c^^,^^d^	210 (49.9)^a^^,^^b^^,^^c^^,^^d^	76 (20.9)^a^^,^^b^^,^^c^
*p*-value		<0.001	<0.001	<0.001	<0.001
Abuse type					
None	1,203	153 (11.0)	19 (1.0)	203 (15.7)	50 (3.9)
Hands-on CSA only	96	32 (25.5)^e^	5 (11.1)^e^	38 (36.9)^e^	11 (22.5)^e^
IBSA only	1,006	295 (22.0)^e^	59 (4.1)^e^	345 (25.4)^e^	95 (8.0)^e^^,^^f^
Both	325	178 (41.7)^e^^,^^g^	52 (10.5)^e^^,^^g^	192 (44.2)^e^^,^^g^	74 (19.2)^e^^,^^g^
*p*-value		<0.001	<0.001	<0.001	<0.001

Row percentages.

a Significantly different from 0 deficits.

b Significantly different from 1 deficit.

c Significantly different from 2 deficits.

d Significantly different from 3 deficits.

e Significantly different from none abuse.

f Significantly different from hands-on abuse only.

g Significantly different from IBSA only.

Abuse type was also significantly associated with all health indicators. Those reporting both CSA and IBSA had the highest rates of both lifetime suicide attempt and prior to the age of 18, and this was significantly different from other types of abuse except hands-on abuse only. Indeed, among those experiencing both abuse types, 41.7% reported suicide attempt before age 18 compared to 22.0% of those with IBSA only, 25.5% of those with CSA only, and 11.0% of those with no abuse (*p* < 0.001). The large difference in percent between those with CSA only (25.5%) and both abuse types (41.7%), however, may be attributable to power issues as the actual cell size for CSA only x suicide attempt before age 18 is small (*n* = 32).

One percent of those with no abuse reported an overdose before age 18, followed by 4.1% of those with IBSA only, 11.1% of those with CSA only, and 10.5% of those with both abuse types (*p* < 0.001). Those with both abuse types and with CSA only had statistically similar proportions of overdose both before age 18 and lifetime.

### Multivariate analyses

#### Predictive margins of outcomes at the intersection of SDoH deficit count and abuse

To examine how SDoH and abuse types interact and their association with suicide and overdose outcomes, we performed a logistic regression of each outcome regressed SDoH deficit count and abuse type, while controlling for age, sexual upon identity, gender identity, race and ethnicity. We then predicted the margins of *SDoH deficit x abuse type*. Table 4 shows the predicted probabilities.

**Table 4:** Predictive margins of suicide and overdose outcomes based on the intersection of the number of SDoH deficits and type of abuse.

Abuse type								
SDoH deficits	None		Hands-on CSA Only		IBSA only		Both CSA and IBSA	
	Margin	95% CI	Margin	95% CI	Margin	95% CI	Margin	95% CI
Suicide attempt before age 18								
0 SDoH	0.09	0.06–0.12	0.21	0.02–0.40	0.15	0.11–0.20	0.25	0.15–0.36
1 SDoH	0.12	0.07–0.18	0.28	0.05–0.50	0.21	0.14–0.28	0.33	0.19–0.47
2 SDoH	0.17	0.10–0.24	0.35	0.10–0.61	0.28	0.17–0.38	0.42	0.26–0.58
3 SDoH	0.14	0.05–0.22	0.30	0.05–0.55	0.23	0.11–0.34	0.36	0.20–0.52
4 + SDoH	0.24	0.14–0.33	0.45	0.17–0.74	0.36	0.25–0.47	0.52	0.38–0.65
Overdose before age 18								
0 SDoH	0.01^ns^	−0.00–0.02	0.09^ns^	−0.07–0.25	0.03	0.01–0.04	0.05	0.01–0.09
1 SDoH	0.01	0.00–0.03	0.16^ns^	−0.07–0.38	0.05	0.00–0.09	0.09	0.01–0.17
2 SDoH	0.01^ns^	−0.00–0.02	0.10^ns^	−0.04–0.24	0.03	0.00–0.05	0.05^ns^	−0.01–0.11
3 SDoH	0.01^ns^	−0.00–0.03	0.15^ns^	−0.05–0.36	0.05	0.01–0.08	0.09	0.02–0.15
4 + SDoH	0.03	0.00–0.06	0.30^ns^	−0.05–0.65	0.10	0.03–0.18	0.18	0.06–0.31
Lifetime suicide attempt								
0 SDoH	0.13	0.09–0.17	0.31	0.10–0.51	0.18	0.13–0.22	0.26	0.16–0.37
1 SDoH	0.19	0.12–0.25	0.41	0.17–0.65	0.25	0.18–0.33	0.36	0.22–0.50
2 SDoH	0.22	0.14–0.30	0.46	0.21–0.71	0.29	0.19–0.40	0.41	0.25–0.56
3 SDoH	0.18	0.09–0.28	0.40	0.15–0.66	0.25	0.13–0.37	0.35	0.20–0.51
4 + SDoH	0.35	0.24–0.46	0.62	0.37–0.86	0.44	0.32–0.55	0.56	0.43–0.70
Lifetime overdose								
0 SDoH	0.03	0.01–0.05	0.17	0.00–0.33	0.06	0.03–0.08	0.10	0.04–0.17
1 SDoH	0.04	0.02–0.06	0.21	0.00–0.41	0.07	0.02–0.12	0.13	0.02–0.24
2 SDoH	0.05	0.01–0.09	0.26^ns^	−0.00–0.51	0.09	0.02–0.16	0.16	0.04–0.29
3 SDoH	0.05	0.00–0.10	0.25^ns^	−0.00–0.51	0.09	0.00–0.18	0.16	0.03–0.30
4 + SDoH	0.10	0.03–0.17	0.41	0.11–0.71	0.17	0.09–0.26	0.29	0.15–0.42

All models adjust for current age, sexual minority status before age 18, gender minority status before age 18, racial minority, ethnic minority, and sex at birth (female vs not). All predicted probabilities are statistically significant at *p* < 0.05 unless otherwise noted (^ns^). SDoH, social determinants of health deficits. CSA, child sexual abuse. IBSA, image-based sexual abuse.

The combination of SDoH deficit and abuse type compounds the increase in probability of suicide attempt before age 18 across each abuse type, including those with no reported abuse (Table 4). The probability of a suicide attempt before age 18 increases from 0.09 (0 SDoH deficits) to 0.24 (4 + SDoH deficits) among those with no reported abuse, from 0.21 to 0.45 among those with CSA only, from 0.15 to 0.36 among those with IBSA only, and from 0.25 to 0.52 among those with both types of abuse, representing the largest increase in predicted probability.

**Figure 1:**
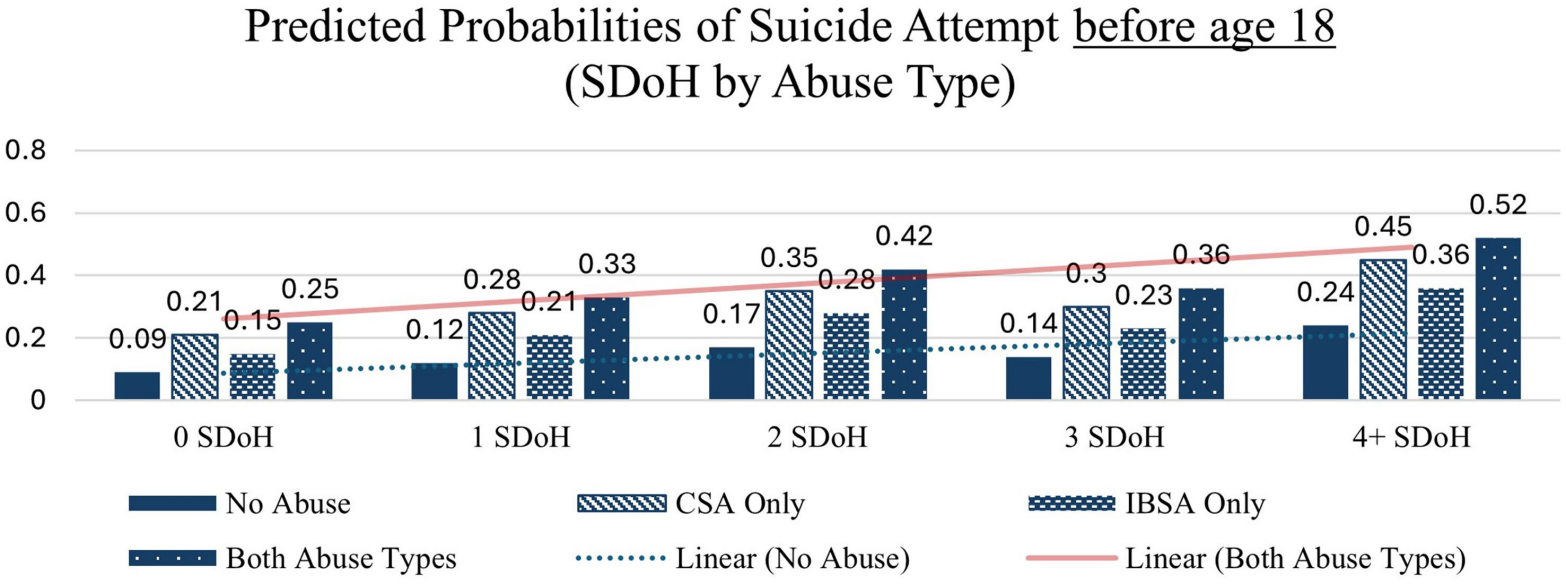
Predicted probabilities of suicide attempt before age 18 (SDoH deficits x abuse type).

**Figure 2:**
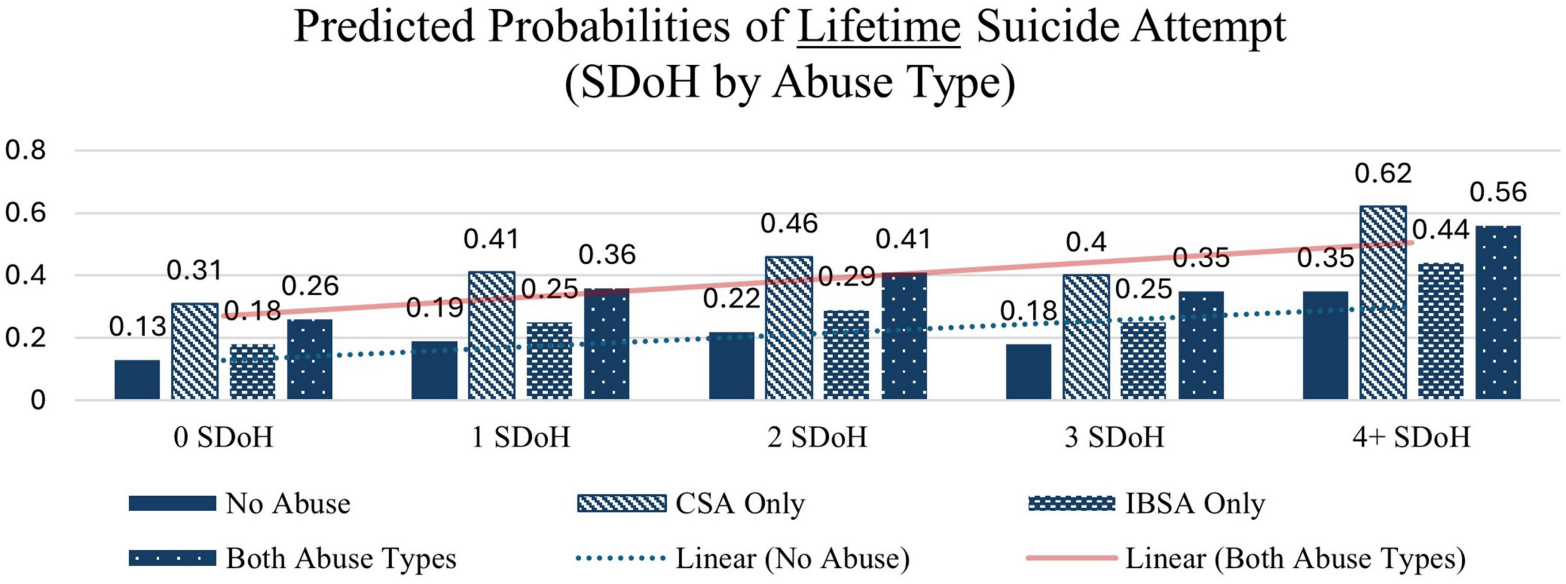
Predicted probabilities of lifetime suicide attempt (SDoH deficits x abuse type).

**Figure 3:**
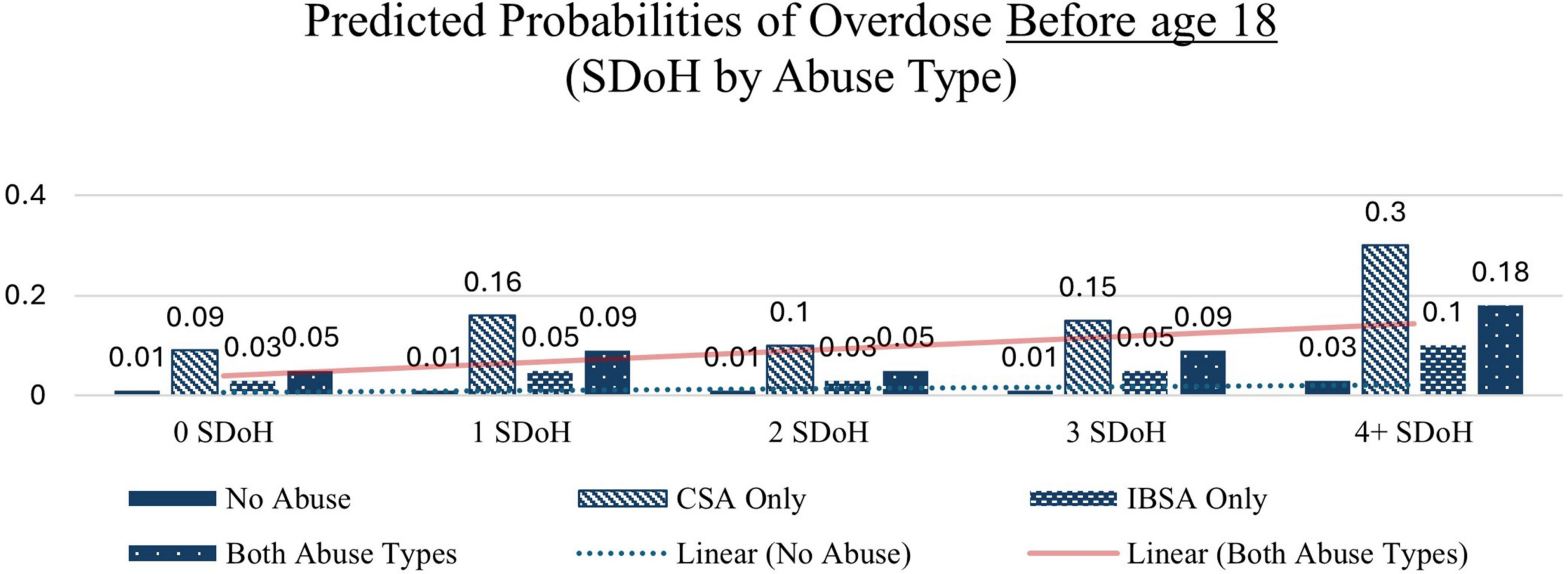
Predicted probabilities of overdose before age 18 (SDoH deficits x abuse type).

**Figure 4:**
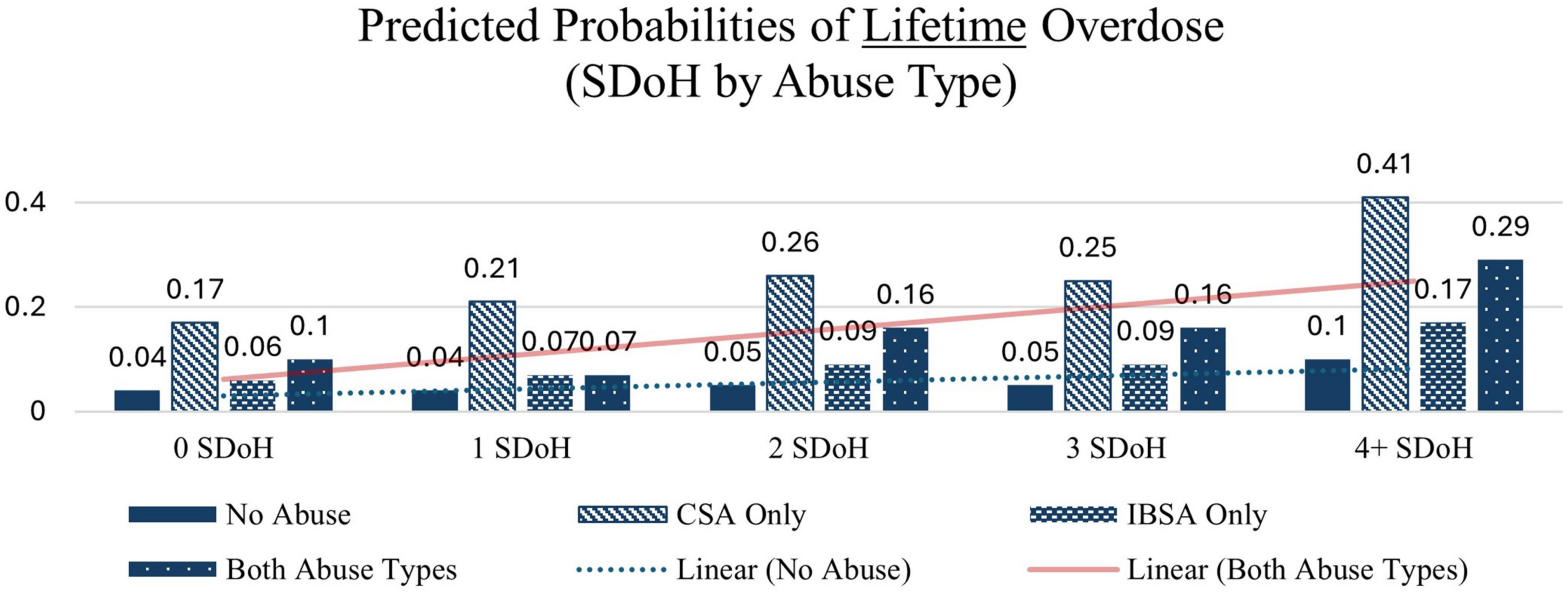
Predicted probabilities of lifetime overdose (SDoH deficits x abuse type).

Similar patterns – with large increases from 0 to 4 + SDoH deficits – are seen across each of the other health indicators except in the case of overdose before age 18 in which the interaction between abuse type and SDoH deficits did not significantly affect probability of overdose before age 18 the none and CSA only abuse types which is likely due to small cell sizes. These interactions are depicted in Figures 1–4.

#### Predictive margins of outcomes at intersection of SDoH domain and abuse

We also examined whether there were differences in the effect of abuse type by each specific SDoH domain. Table 5 shows results of the predictive margins of each health outcome at the intersection of each SDoH domain (economic, social, housing instability, healthcare, and lived environment) and abuse type. These models again control for age, sexual identity, gender identity sex at birth, race, and ethnicity.

**Table 5:** Predictive margins of suicide and overdose on type of SDoH deficit and type of abuse.

Abuse type								
SDoH deficits	None		Hands-on CSA Only		IBSA only		Both CSA and IBSA	
	Margin	95% CI	Margin	95% CI	Margin	95% CI	Margin	95% CI
Suicide attempt before age 18								
Economic	0.13	0.09–0.18	0.27	0.06–0.49	0.21	0.15–0.27	0.33	0.22–0.45
Social	0.22	0.15–0.30	0.42	0.15–0.70	0.34	0.25–0.42	0.50	0.36–0.63
Housing	0.15	0.07–0.23	0.30	0.04–0.57	0.23	0.12–0.35	0.37	0.20–0.53
Healthcare	0.09	0.05–0.14	0.20	0.02–0.38	0.15	0.09–0.21	0.25	0.15–0.35
Environment	0.15	0.09–0.20	0.30	0.07–0.53	0.23	0.16–0.30	0.36	0.22–0.50
Overdose before age 18								
Economic	0.02	0.00–0.04	0.16^ns^	−0.08–0.40	0.06	0.01–0.11	0.11	0.04–0.18
Social	0.02^ns^	−0.00–0.04	0.14^ns^	−0.06–0.34	0.05	0.02–0.09	0.09	0.03–0.16
Housing	0.01^ns^	−0.00–0.03	0.11^ns^	−0.09–0.30	0.04	0.01–0.07	0.07	0.00–0.14
Healthcare	0.02^ns^	−0.00–0.05	0.18^ns^	−0.08–0.44	0.07	0.02–0.13	0.12	0.04–0.21
Environment	0.01^ns^	−0.00–0.02	0.08^ns^	−0.05–0.22	0.03	0.01–0.05	0.05	0.02–0.09
Lifetime suicide attempt								
Economic	0.19	0.14–0.24	0.39	0.15–0.62	0.25	0.18–0.31	0.36	0.24–0.47
Social	0.29	0.21–0.37	0.53	0.28–0.78	0.37	0.28–0.45	0.49	0.36–0.63
Housing	0.23	0.12–0.34	0.45	0.17–0.72	0.30	0.17–0.43	0.41	0.24–0.58
Healthcare	0.16	0.09–0.22	0.33	0.11–0.54	0.20	0.13–0.28	0.30	0.19–0.41
Environment	0.21	0.14–0.28	0.41	0.17–0.66	0.27	0.20–0.34	0.38	0.24–0.52
Lifetime overdose								
Economic	0.06	0.03–0.10	0.27	0.02–0.53	0.11	0.05–0.17	0.19	0.09–0.28
Social	0.06	0.02–0.10	0.25	0.03–0.48	0.10	0.04–0.16	0.17	0.07–0.27
Housing	0.05	0.01–0.09	0.22^ns^	−0.01–0.46	0.09	0.03–0.14	0.15	0.05–0.25
Healthcare	0.07	0.02–0.11	0.27	0.03–0.51	0.11	0.05–0.17	0.19	0.08–0.29
Environment	0.04	0.02–0.07	0.20^ns^	−0.01–0.41	0.08	0.04–0.11	0.13	0.03–0.23

All models adjust for current age, sexual minority status before age 18, gender minority status before age 18, racial minority, ethnic minority, and sex at birth (female vs not). All predicted probabilities are statistically significant at *p* < 0.05 unless otherwise noted (^ns^). SDoH, social determinants of health deficits. CSA child sexual abuse. IBSA, image-based sexual abuse.

For all SDoH domains, the predictive margins of suicide attempt before age 18 were highest among individuals who reported experiencing both abuse types. For example, the predicted margins of suicide attempt before age 18 was 0.15 (95% CI = [0.09–0.20]) for those with no reported abuse, compared to 0.36 [0.22–0.50] for those who reported both types of abuse within the domain of environmental SDoH deficit and 0.22 [0.15–0.30] for those with no abuse compared to 0.50 [0.36–0.63] for those with both abuse types within the domain of social SDoH deficit.

## Discussion

This study examined links between IBSA and CSA and past and lifetime key public health indicators – suicide and drug overdose. Among participants in this study which were purposely screened to maximize the inclusion of those having childhood IBSA experiences, 38.3% reported childhood IBSA only (with no reported hands-on CSA), 3.7% reported hands-on CSA without IBSA, and 12.4% reported both experiences. Importantly, the high co-occurrence of CSA and IBSA—where over 77% of CSA survivors also reported experiencing IBSA—suggests that these two forms of abuse are often intertwined rather than distinct victimization experiences. On the other hand, a notable majority of experiences involved only IBSA without any hands-on CSA offense. This suggests wide diversity in the types of IBSA incidents youth experience going beyond child sexual abuse with production that has dominated the field prior to the widespread use of technology among today’s youth and adults.

### Mental health indicators in relation to CSA and IBSA

Our findings reinforce prior research indicating that CSA is significantly associated with adverse health indicators, including suicide attempts and substance use disorders (9, 11, 42). However, this study extends the literature by showing that IBSA is similarly associated with these indicators, with relationships comparable to those observed in hands-on CSA. While IBSA can occur independently, the overlap observed in this study highlights how youth subjected to hands-on CSA are also at heightened risk for IBSA. This pattern underscores the need to conceptualize IBSA as part of a broader continuum of sexual exploitation rather than treating it as an isolated phenomenon. The presence of both abuse types was associated with the highest percentage of suicide attempt before age 18, and, although not statistically significant at the multivariate level, was noticeably larger (41.7% both those experiencing both IBSA and CSA vs. 25.5% with CSA only); this was likely due to a lack of statistical power given the small number of participants with a suicide attempt prior to age 18 that reported *only* hands-on CSA (*n* = 32). Although more research is needed, findings offer some early indication that cumulative victimization may amplify psychological distress and increase vulnerability to self-harm when occurring contemporaneously (that is, during childhood). These findings align with poly-victimization theory (43, 44) which posits that exposure to multiple forms of abuse exacerbates mental health risks by reinforcing learned helplessness, increasing emotional dysregulation, and diminishing protective coping mechanisms. This also supports the adverse childhood experiences literature which highlights the particularly damaging health impact of experiencing multiple adversities in childhood (45, 46).

### The role of social determinants of health

The findings emphasize the importance of extending the field’s conceptualization of IBSA, CSA and mental health to include the contribution of SDoH. Rather than focusing exclusively on demographic variables in understanding these relationships, this study examines potentially modifiable social and structural variables that have been connected to health outcomes. Indeed, data from this study underscores the importance of taking into account SDoH when addressing and screening for suicide and overdose outcomes in both adolescence and young adulthood. The high percentages of negative health indicators among participants reporting both CSA and IBSA may be a product of simultaneously experiencing higher SDoH deficits. Indeed, higher SDoH deficits were independently associated with increased suicide and overdose risk, even in the absence of abuse history. Specifically, youth with four or more SDoH deficits had significantly higher predicted probabilities of suicide attempts and overdoses compared to those with fewer or no deficits. These results suggest that structural disadvantages can amplify the psychological impact of CSA and IBSA, further increasing the likelihood of adverse health outcomes.

The accumulation of SDoH across multiple sectors, including economic instability, discrimination, housing insecurity, and limited healthcare access appears to compound psychological distress, further exacerbating risk among youth who have also experienced sexual victimization. These findings extend our knowledge by highlighting the compounding impact of experiencing different childhood sexual abuse and exploitation experiences while also experiencing different levels of SDoH deficits. Indeed, although young adults with histories of sexual abuse were more likely to have poorer mental and behavioral health indicators, both before and after age 18, this was particularly true for those same youth who also experienced increasing numbers of SDoH deficits. These results are consistent with growing research in the field about links between discrimination and health (47) and suicide behaviors in particular (48), demonstrating that structural disadvantages contribute to poor mental health by limiting access to protective resources, increasing exposure to chronic stressors, and reinforcing cycles of social marginalization. Overall, those with both IBSA and hands-on CSA were the most likely to report each type of SDoH as well as more overall. This supports prior research that highlights the relationship between risk factors across multiple levels of social ecology and child health (49).

### Implications for prevention and intervention

This study revealed several key findings that have implications for both abuse intervention and mental and behavioral health prevention programs. First, IBSA is linked with CSA in important ways that could help CSA investigations. Although the current data cannot differentiate between IBSA that was specifically part of CSA or whether it was a separate incident, it does suggest the high likelihood of youth experiencing CSA to also having experienced IBSA. CSA investigations conducted by multi-disciplinary teams and law enforcement should always consider the existence of IBSA as it not only has implications for victim impact, but also could serve as concrete evidence in cases rather than relying on victim testimony.

Second, suicide and overdose screening efforts must incorporate both SDoH and abuse histories. Our findings indicate that relying solely on SDoH screenings without considering CSA or IBSA could miss at-risk youth. Likewise, screening solely for abuse history without assessing social determinants may overlook the compounded vulnerabilities that arise from structural disadvantages. As noted by Karatekin et al. (50) in a scoping review on Adverse Childhood Experiences, research often focuses on the “downstream” factors of victimization and adversity rather than the structural, modifiable “upstream” factors and interventions occurring outside of the home. Given the demonstrated cumulative effect of SDoH deficits, intervention strategies must address these broader systemic issues rather than focusing exclusively on individual risk factors.

Third, survivors of IBSA may require specialized interventions that account for the ongoing fear of image circulation, public exposure, and social stigmatization reported by some survivors. Findings from the current analyses suggest the combination of both CSA and IBSA may compound health indicators for some youth. This, combined with research documenting the unique mental health impact of IBSA, even in the context of CSA (7), suggests IBSA victimization may extend beyond the initial event for some youth, with long-term consequences that can shape identity, self-esteem, and mental health. Future research should examine how IBSA-specific interventions can mitigate these enduring harms.

### Future research directions

Future research needs to better understand when and how IBSA is part of a specific CSA victimization, and how this impacts mental health. Indeed, survivors of IBSA often report feeling ongoing fear over the circulation or resurfacing of their images online, as well as worry about being recognized in public. The sharing of images and the public accessibility of the images is one of the most difficult aspects of the crime to overcome (51, 52), and contributes to the feelings of ongoing vulnerability, helplessness and powerlessness (52–54).

There is also a need for longitudinal research which explores the long-term implications of IBSA, particularly in comparison to hands-on CSA. Prior research has established long-term negative outcomes linked with CSA (10, 55), but little is known about how IBSA interacts with these effects over time. Understanding the interplay between these abuse types and SDoH can inform more comprehensive prevention and intervention efforts. Additionally, qualitative research is needed to capture the nuanced experiences of IBSA survivors. Future studies should examine how survivors navigate ongoing digital threats, social stigma, and emotional distress related to their victimization. Exploring these lived experiences can help tailor interventions to better support those affected by IBSA and CSA. Finally, more research is needed to better understand the range of IBSA experiences that occur which do not involve hands-on CSA. These incidents were commonly reported in this sample. The diversity of these cases likely require different approaches to prevention and intervention.

### Limitations

All of the constructs in this study were measured retrospectively and focused on experiences prior to the age of 18. As such, causal relationships cannot be determined. Sampling bias could exist due to the opt-in recruitment methods via social media, although this was less of a concern given ads did not reveal study aims. Moreover, it is possible that our screening method did not capture those with past IBSA experiences who may have different mental health profiles than those who enrolled in the study. That is, it is possible that some with IBSA victimization would answer the screener questions with “1” (not at all likely), given they are now more wary of sharing sexual photos or videos of themselves because of their victimization experience. These individuals may look different, with respect to health indicators, than eligible study participants. This is an important consideration for future research aiming to measure IBSA and related experiences. The key variables, including hands-on CSA, drug overdose, and IBSA could be part of the same episode; we were unable to separate these out as unique experiences due to the structure of the survey. Moreover, our measurement of hands-on CSA only included adult perpetrators. It is possible that some of those coded into the “No abuse” category did, in fact, experience sexual abuse by a sibling or peer. This was beyond the scope of this study, and additional research should examine these episodes in the context of abuse experiences and mental health indicators.

Fraud is always a concern with social media recruitment (56), however, we included extensive procedures to deter and detect any fraudulent entries; steps designed and tested by the authors across multiple studies which reduces concerns in this regard (7). A further limitation is that we were unable to separately examine adulthood (after age 18) health indicators from overall lifetime measures due to the low endorsement rates of after age 18 *only* suicide attempts and overdoses in our sample. This means that while we could assess suicide attempts and overdoses that occurred before age 18, our lifetime measures include both adolescent and adult experiences, preventing a clear understanding of how CSA, IBSA, and SDoH deficits uniquely affect health beyond adolescence. Finally, our measures of suicide attempt did not capture important details, like age of onset, frequency and duration, that would be helpful for practitioners in assessing risk level.

## Conclusion

This study underscores the compounded risks faced by youth exposed to CSA, IBSA, and SDoH deficits. Findings suggest that trauma and structural adversity are interwoven factors that jointly shape long-term health trajectories. Addressing these challenges requires integrated screening strategies, trauma-informed interventions, and systemic policy efforts that target both individual, social and structural risk factors. By shifting from a purely individualistic model of risk assessment to a more holistic framework—one that acknowledges both trauma and structural barriers—healthcare providers, policymakers, and researchers can better support vulnerable youth and reduce the long-term burden of suicide and substance use.

## Data Availability

The dataset presented in this article are not readily available because data will be archived at ISPCR at the end of the project. Requests to access the dataset should be directed to KM, kimberly.mitchell@unh.edu.

## Footnotes

^1^We did not consider interactions between sexual identity and gender identity when designing our survey weights, as it was not clear that we would have weighting targets at that level and to avoid adding more complexity to already strenuous weighting. See Silva and Evans (57) and Herman et al. (58) for sources on interactions between sex and gender identity.
