# Supplementary material for: Cumulative determinants of adolescent health indicators: the effects of social and structural determinants of health and child sexual abuse on overdose and suicide attempt
Source: Front Public Health. 2025 Jun 27;13:1595115. doi: 10.3389/fpubh.2025.1595115 (PMC12245784; doi:10.3389/fpubh.2025.1595115)
Supplement: Supplementary file 1 [file Table_1.docx]

| **Supplementary Table 1. Sample demographic characteristics.** | | |
| --- | --- | --- |
| Demographic Characteristic | Unweighted n (%) | Weighted % |
| Female at birth | 1911 (72.7) | 61.2 |
| Sexual identity minority before age 18 | 1446 (56.4) | 21.3 |
| Gender minority identity before age 18 | 517 (19.7) | 4.8 |
| Racial minority identity | 774 (29.4) | 31.5 |
| Ethnic minority identity | 423 (16.1) | 19.1 |
|  | Mean (Standard Deviation) | Mean (Standard Error) |
| Current age (years) | 22.32 (3.14) | 23.0 (0.10) |

**Supplemental Table 2. Original and dichotomous SDoH deficit items**

| **Type of SDoH** | **Original item wording** | **Original scale item** | | **Recoded dichotomous item** | **Threshold of new item** |
| --- | --- | --- | --- | --- | --- |
|  |  | **M (SD)** | **Range** | **Unweighted n (%)** |  |
| **Economic stability** |  |  |  |  |  |
| Not enough money to pay the bills | “How often did this describe you or your family before you turned 18? We did not have enough money to pay the bills.” | 1.13 (1.22) | 0 – 4 | 468 (17.79) | >1sd above mean |
| Cell phone turned off | “Before you turned 18, how often has your cell phone been turned off because you or your family did not have enough money to pay the bill.” | 0.41 (0.91) | 0 – 4 | 375 (14.26) | >1sd above mean |
| Skipped meals | “Before you turned 18, how often did you skip meals or eat less because you or your family didn’t have enough money for food?” | 0.65 (1.03) | 0 – 4 | 548 (20.84) | >1sd above mean |
| **Social context** |  |  |  |  |  |
| Non-victimization adversity | “Now I’d like to ask you about some other experiences that **you or someone in your family** may have had before you were 18 years old” | 3.31 (2.24) | 0 – 11 | 452 (17.19) | >1sd above mean |
| Discrimination | “In your day-to-day life **before turning 18**, how often did any of the following things **happen to you**?” | 14.81 (11.02) | 0 – 45 | 500 (19.01) | >1sd above mean |
| **Healthcare** |  |  |  |  |  |
| Last time saw dentist - n(%) | “Before you were 18, how often did you see a dentist for a check-up, exam, teeth cleaning, or other dental work? Your best guess is fine” |  |  |  |  |
| Once a year  Once every 2-3 years  Once every 4-5 years  Once or twice before I turned 18  I never went to the dentist before I turned 18 |  | 1.52 (0.93) | 1 – 5 | 343 (13.04) | Less often than once every 4-5 years |
|  |  |  |  |  |  |
|  |  |  |  |  |  |
|  |  |  |  |  |  |
| **Neighborhood and built environment** |  |  |  |  |  |
| Unstable housing | “What about before you turned 18 years old, did you experience any of the following sleeping situations because **you did not have a permanent place to stay?”** | 1.05 (0.49) | 0 – 7 | 163 (6.20) | >1sd above mean |
| Residence problems | “Now, please think about where you **lived for most of the time before you turned 18.** Were there any of these problems where you live? Please think about your permanent place of residence before turning 18, not a dorm room or other temporary housing (*mark all that apply).”* | 0.96 (1.52) | 0 – 8 | 404 (15.36) | >1sd above mean |
| Neighborhood problems | “Please tell me if each of the following was a serious problem, minor problem, or no problem at all in your neighborhood… by neighborhood we mean the street you lived on and a few streets around it.” | 3.95 (3.91) | 0 – 12 | 450 (17.11) | >1sd above mean |

SDoH = social determinants of health
